# Targeting the Integrated Stress Response Kinase GCN2 to Modulate Retroviral Integration

**DOI:** 10.3390/molecules26175423

**Published:** 2021-09-06

**Authors:** Chloé Torres, Asja Garling, Saïd Taouji, Christina Calmels, Marie-Line Andreola, Mathieu Métifiot

**Affiliations:** 1 Univ. Bordeaux, CNRS, UMR 5234, Microbiologie Fondamentale et Pathogénicité, F-33076 Bordeaux, France; chloe.torres@etu.u-bordeaux.fr (C.T.); Asja.Garling@hotmail.com (A.G.); christina.calmels@u-bordeaux.fr (C.C.); marie-line.andreola@u-bordeaux.fr (M.-L.A.); 2 Actions for onCogenesis Understanding and Target Identification in ONcology (ACTION), UMR 1218, Bergonie Institute, F-33076 Bordeaux, France; said.taouji@inserm.fr

**Keywords:** high-throughput screening, protein–protein interaction, integrated stress response, HIV integration, AlphaLISA, drug repurposing, assay development

## Abstract

Multiple viral targets are now available in the clinic to fight HIV infection. Even if this targeted therapy is highly effective at suppressing viral replication, caregivers are facing growing therapeutic failures in patients due to resistance, with or without treatment-adherence glitches. Accordingly, it is important to better understand how HIV and other retroviruses replicate in order to propose alternative antiviral strategies. Recent studies have shown that multiple cellular factors are implicated during the integration step and, more specifically, that integrase can be regulated through post-translational modifications. We have shown that integrase is phosphorylated by GCN2, a cellular protein kinase of the integrated stress response, leading to a restriction of HIV replication. In addition, we found that this mechanism is conserved among other retroviruses. Accordingly, we developed an in vitro interaction assay, based on the AlphaLISA technology, to monitor the integrase-GCN2 interaction. From an initial library of 133 FDA-approved molecules, we identified nine compounds that either inhibited or stimulated the interaction between GCN2 and HIV integrase. In vitro characterization of these nine hits validated this pilot screen and demonstrated that the GCN2-integrase interaction could be a viable solution for targeting integrase out of its active site.

## 1. Introduction

HIV therapy has tremendously improved over the past two decades. All three virally encoded enzymes, reverse-transcriptase, integrase (IN) and protease, have been targeted by small molecule inhibitors [1]. There are still a few limitations to be addressed, such as resistance and treatment adherence due to the poor pharmacodynamic properties of first-generation drugs. Today, a new generation of molecules have improved properties and can even achieve several weeks of efficacy, depending on their administration route (long-lasting strategies). However, resistance phenomena still have not been overcome, and the transmission of multi-resistant strains of the virus leaves a growing number of patients without any solution [2]. Hence, we are in the urgent need of developing innovative therapeutic targets.

HIV-1 IN catalyzes the integration of viral DNA into the cellular genome [3]. IN is part of a pre-integration complex composed of cellular and viral proteins [4]. The dynamic of these interactions regulates IN activities but also noncatalytic activities, such as nuclear import and tethering of the complex to the integration site. Studies have provided important insights into the post-translational modifications (PTM) of IN, which regulate its multifaceted functions [5]. Yet, IN’s functions are fine-tuned by these PTMs is undetermined. In a previous work, we identified GCN2 as a cellular partner of IN via a yeast two-hybrid system [6]. GCN2 is a cellular protein kinase involved in stress responses, along with PERK, PKR and HRI [7]. Different stresses have been shown to activate GCN2, such as amino acid starvation and UV irradiation. Upon autophosphorylation (p-GCN2), p-GCN2 phosphorylates eIF2α, which results in the control of translation. While this control of translation happens in the cytosol, a recent study showed that nutrient starvation induced a nucleolar localization of GCN2 [8]. GCN2 has been implicated in human burdens such as cancer and Alzheimer’s disease and in addition, we showed that GCN2 is activated during HIV-1 infection [9].

In vitro, phosphorylation assays using recombinant enzymes established that GCN2 efficiently phosphorylates IN from HIV-1 but also from other retroviruses, such as MLV and ASV [10]. In cells, depletion of GCN2 strikingly increased infectivity of HIV-1, establishing a link between GCN2 and retroviral replication. In agreement, infectivity of HIV-1 was also increased in the context of viruses harboring IN mutations unable to sustain phosphorylation by GCN2. Although reverse transcription was not affected, integration was increased in these mutant viruses.

A tremendous effort has been made to discover effective kinase inhibitors in the past couple decades [11]. Most of them are active-site inhibitors, with limited selectivity, leading to multiple kinase inhibition (polypharmacology). This property also raised concerns about off-target events potentially leading to long-term adverse effects, such as genome instability. Accordingly, more selective compounds are being developed, including molecules targeting protein–protein interactions instead of the kinase-active site [12]. Targeting protein–protein interactions is a growing field of research, including in both in vitro and in silico studies [13,14,15,16]. This is true in the context of kinases—as with Bcl-2, for example—but it is also true in the context of HIV research and IN. The case of LEDGF/p75 has proved that not only IN activity mattered, but also the overall shape of the enzyme. Interfering with the oligomerization of IN through the IN-LEDGF interaction interface induced profound morphological changes in HIV progenies that were then intrinsically inactivated [17]. In this study, we set up an in vitro assay to monitor the IN–GCN2 interaction using AlphaLISA. Following optimization, a pilot screen was conducted to validate the assay. We identified nine molecules able to modulate the IN–GCN2 interaction. Further characterization is needed to determine whether these FDA-approved drugs may be repurposed as anti-HIV agents, but, altogether, we demonstrate that the IN–GCN2 interaction can be targeted by small molecule ligands.

## 2. Results

### 2.1. Setup of the IN–GCN2 Interaction Assay

**Figure 1:**
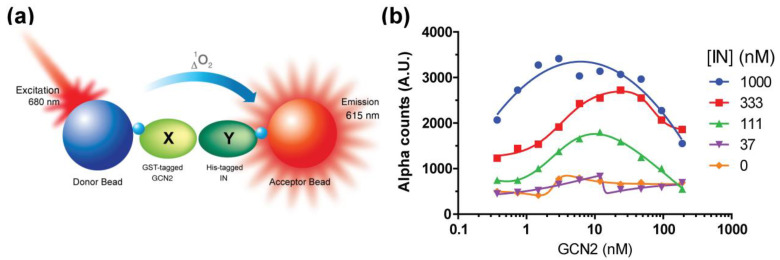
(**a**) Protein–protein interaction assay principle using AlphaLISA. (**b**) Cross-titration experiment.

We developed an in vitro plate-based assay to monitor the interaction between GCN2 and IN using the AlphaLISA technology (Figure 1). Taking advantage of the tags present on each recombinant protein (6xHis for IN prepared “in-house”, and GST for GCN2 commercially available), we used specific beads that fluoresce only when they are in close proximity (<200 nm). The signal obtained (light count) is directly proportional to the binding of IN to GCN2 (homogenous solution assay at steady state). Cross-titration experiments showed that the increase in IN concentration up to 1 µM was associated with an increase in signal (Figure 1a). On the other hand, increasing the concentration of GCN2 resulted in a characteristic hook effect, with an optimum reached between 5 and 50 nM. Accordingly, we decided to perform all further experiments at 25 nM and 700 nM for GCN2 and IN, respectively that correspond to the experimental conditions used in the in vitro phosphorylation assay [10].

**Figure 2:**
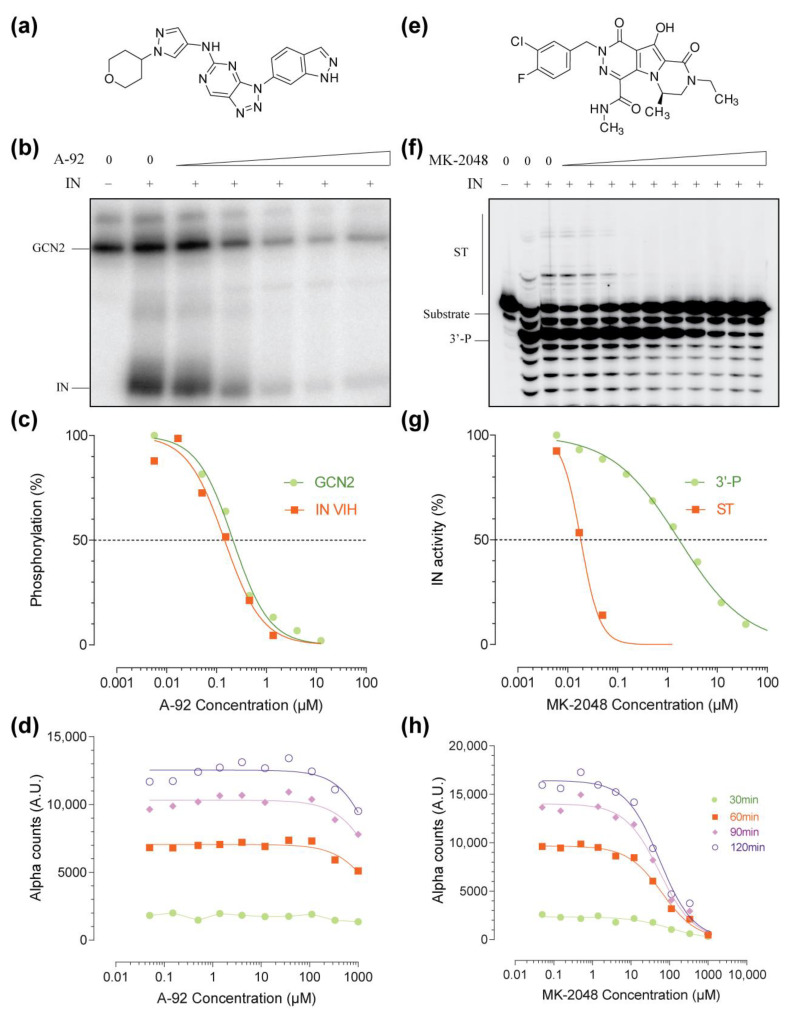
Effect of active site inhibitors on proteins’ activities and interaction. Chemical structure of A-92 (**a**) and MK-2048 (**e**); autoradiography of a representative in vitro phosphorylation assay (**b**) or integration assay (**f**) using increasing concentration of active site inhibitors. Concentrations of A-92 and MK-2048 were from 3.7 µM to 45.7 nM and from 111 µM to 5.6 nM, respectively, using a 3-fold serial dilution. Quantification of GCN2’s activation (auto-phosphorylation) and phosphorylation of IN (**c**) and quantification of the 3′-P and ST activity (**g**) in the presence of increasing concentrations of inhibitor. Monitoring of the IN–GCN2 interaction over time in the presence of increasing concentrations of A-92 (**d**) or MK-2048 (**h**).

A-92 is a specific active site inhibitor of GCN2 (Figure 2a) [18,19]. In vitro, A-92 inhibited both GCN2’s activation (autophosphorylation) and IN’s phosphorylation catalyzed by GCN2 (Figure 2b). Both activities were similarly affected with an apparent IC_50_ of around 200 nM (Figure 2c). When used in the AlphaLISA assay, A-92 did not affect the IN–GCN2 interaction over a wide concentration range and only high doses above 333 µM had a slight impact on interaction (less than 20% inhibition at 1 mM of A-92, Figure 2d).

MK-2048 is a specific IN active site inhibitor (Figure 2e) [20]. Developed by Merck as a second-generation IN inhibitor prototype, it exhibits high selectivity for the inhibition of the strand transfer reaction (ST) over the 3′-processing (3′-P, Figure 2f). This selectivity is exemplified in the gel-based assay by the inhibition of ST at low doses of MK-2048 (nanomolar range) while micromolar doses of MK-2048 were required to inhibit the 3′-P (Figure 2g). Consistent with the literature, the apparent IC_50s_ were 20 nM and 2 µM for the inhibition of ST and 3′-P, respectively. At either nanomolar or low micromolar ranges, MK-2048 did not affect the IN–GCN2 interaction (Figure 2h). Still, a complete inhibition could be achieved in the millimolar range, with an apparent IC_50_ of around 100 µM (Figure 2h).

As seen with both A-92 and MK-2048, the trend of the IN–GCN2 interaction is similar at all time-points (Figure 2d,h). Still, the 120 min time point presented a higher dynamic range (S/B ~ 2 Log_10_). Altogether, incubation time did not affect the interaction/inhibition profile and times between 30 min and 120 min are suitable for both monitoring and screening.

### 2.2. Pilot Screen Using a Small Library

**Figure 3:**
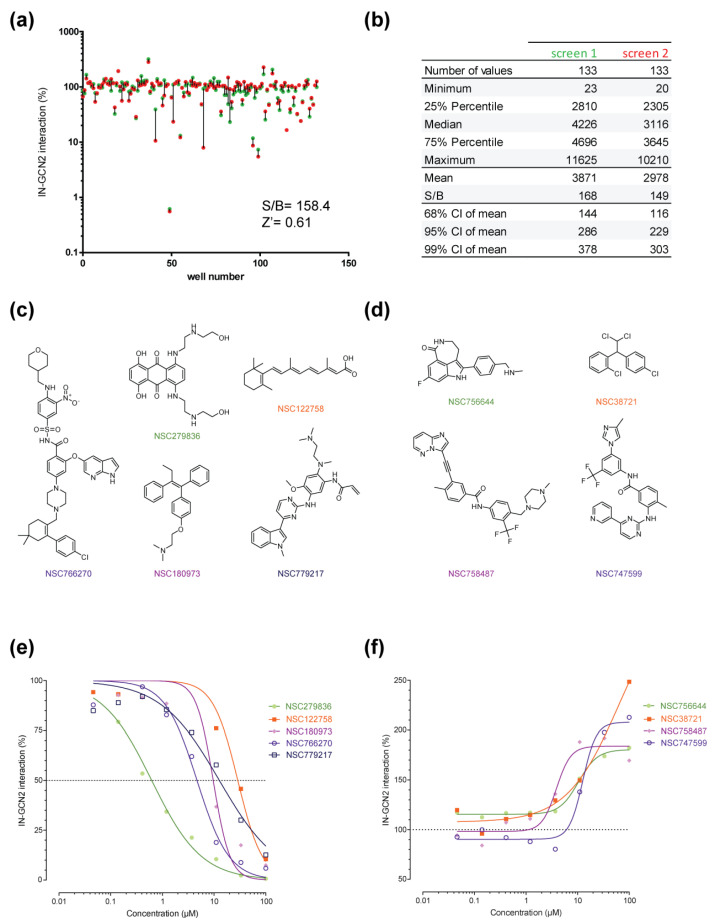
Screening of the DTP oncology set. (**a**) Effect of FDA-approved anticancer drugs on the IN–GCN2 interaction monitored using AlphaLISA. Each compound was tested at a single dose of 100 µM. The plate design was changed to reproduce the experiment and the two independent replicates are represented as red and green dots, linked by a straight vertical line. (**b**) Assay diagnostics. (**c**,**d**) Chemical structure of the best five inhibitors (**c**) and four stimulators (**d**) of the IN–GCN2 interaction. (**e**,**f**) Dose-response curves obtained with either the inhibiting (**e**) or the stimulating compounds (**f**).

To validate the assay, we performed a pilot screen with a small library of molecules. The DTP oncology set is a 133-drug set that have FDA-approval for cancer. These molecules are well characterized, having tremendous data on their bioavailability, toxicity and mechanisms of action in cells. All molecules were initially tested at a single dose of 100 µM (Figure 3a, green dots). From this experiment, we could determine that the assay was robust with a signal to background ratio above 2 Log10, a Z’ of 0.61 (Figure 3a). Next, to ascertain this result and make sure that no technical bias was introduced, we repeated the entire screen following a completely different plate design (Figure 3a, red dots). As seen in Figure 3a, the result was highly reproducible with only few discrepancies (to facilitate comparison, red and green dots corresponding to a single molecule are tied together by a straight line). Additional diagnostic values including minimal and maximal AS counts show that the assay was robust and reproducible (Figure 3b). Altogether, five molecules reproducibly inhibited more than 25% of the signal. The best inhibitor was NSC279836 with 99.4% inhibition at 100 µM followed by NSC766270, NSC180973, NSC122758 and NSC779217 at 93.6%, 89.8%, 87.3% and 83.5%, respectively. No structural similarities could be observed a priori except for NSC766270 and NSC779217 both sharing an indole substitution (Figure 3c). On the other side, four drugs induced an increase in signal of at least two-fold compared to the DMSO control. NSC747599, NSC756644, NSC758487 and NSC38721 induced an increase in signal of 190.1%, 193.2%, 197.2% and 299.9%, respectively. Once again, no structural pattern could be observed within the stimulators (Figure 3d). Of note, NSC758487 presented an indole-like substitution as seen with the two inhibitors NSC766270 and NSC779217.

### 2.3. Hits Validation and Characterization

First, we performed dose-response experiments using the five selected inhibitors and the four stimulators (Figure 3e–f). Starting from the initial screened dose of 100 µM, a 3-fold serial dilution over eight concentrations was performed. In these conditions, the five inhibitors were found to have apparent IC_50_ values ranging from 600 nM to 30 µM, while the stimulators had apparent EC_50_ values ranging from 4 to 60 µM (Figure 3e–f). Interestingly, looking at the highest dose of 100 µM, values obtained in the dose-response experiment were similar to those obtained in both single-dose screen [e.g., 248.5% for NSC38721 and 10.5% for NSC122758 (corresponding to 89.5% inhibition), compare Figure 3a–e,f, respectively).

**Figure 4:**
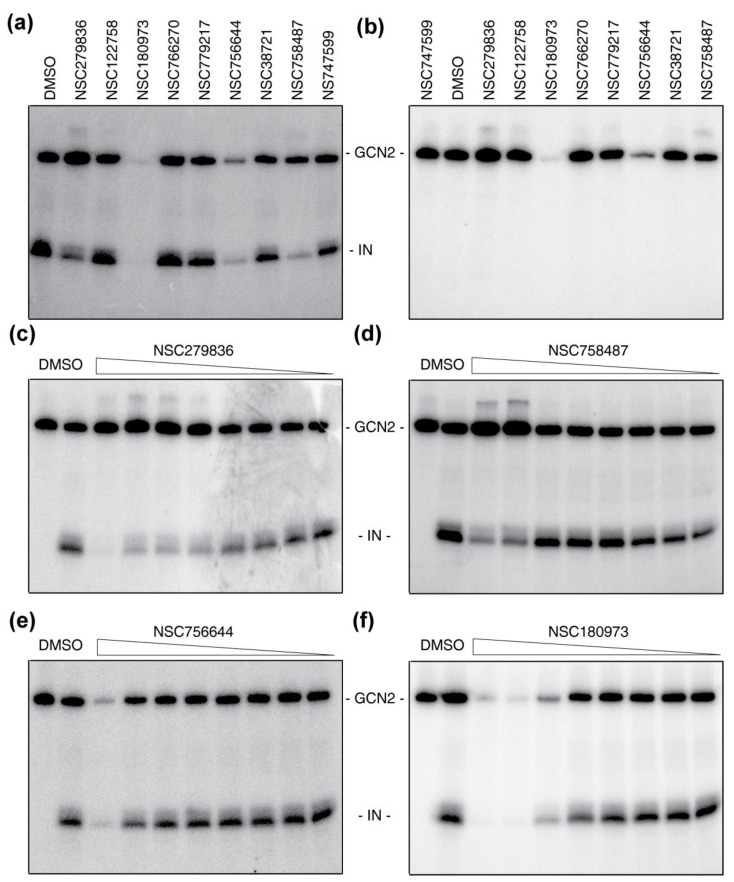
Effect of modulators on GCN2 and IN activities. (**a**) Effect of modulators on IN’s phosphorylation catalyzed by GCN2. Molecules were used at 100 µM corresponding to the initial screen concentration. (**b**) Effect of modulators on GCN2 auto-phosphorylation. The assay was conducted exactly as in panel (**a**) only without IN. Dose-response of active compounds in the phosphorylation assay. Representative images of SDS-PAGE resulting from an in vitro phosphorylation assay of IN catalyzed by GCN2 in the presence of NSC279836 (**c**), NSC758487 (**d**), NSC180973 (**e**), NSC756644 (**f**). First lane corresponds to GCN2 alone while lane two is with IN, both in the presence of DMSO as controls. Lanes three to ten correspond to the conditions with both IN and GCN2 in the presence of decreasing concentrations of molecules from 300 µM to 137 nM, following a 3-fold serial dilution.

Next, we tested all nine modulators for their capacity to affect the phosphorylation of IN catalyzed by GCN2. At the single dose of 100 µM, the two weakest inhibitors NSC122758 and NSC766270 had no impact on the phosphorylation level of neither IN nor GCN2 (compare Figure 4a lanes 1, 3 and 5 and Figure 4b lanes 2, 4 and 6). Regarding NSC779217 and NSC38721, a slight decrease in IN’s phosphorylation could be observed associated to a slight decrease in GCN2 auto-phosphorylation levels (around 20% and 30% decrease respectively, for both IN and GCN2, Figure 4a, lane 6 and Figure 4b, lane 7). Ultimately, both the phosphorylation of IN and GCN2 were inhibited in the presence of 100 µM of NSC180973 and NSC756644 with a decrease of about 90% and 80% in GCN2 auto-phosphorylation, respectively (Figure 4a, lanes 4 and 7 and Figure 4b, lanes 5 and 8). Interestingly, NSC279836 inhibited the phosphorylation of IN (around 65% inhibition) without inhibiting the auto-phosphorylation of GCN2. Furthermore, the signal associated to GCN2 auto-phosphorylation was reproducibly increased in the presence of the inhibitor, with or without IN (170.8% and 144.8% of the DMSO control, respectively, compare Figure 4a lanes 1 and 2 and Figure 4b lanes 2 and 3). Conversely, NSC747599 had no effect on GCN2 auto-phosphorylation (within 10% of the DMSO control) but affected partially IN phosphorylation (40% decrease, Figure 4a lane 10). Finally, NSC758487 slightly affected GCN2 auto-phosphorylation (around 35% decrease compared to DMSO) but drastically impaired IN phosphorylation (>80% decrease compared to DMSO, Figure 4a lane 9).

These effects were confirmed using serial dilutions of NSC279836, NSC758487, NSC180973 and NSC756644 from 300 µM to 137 nM following 3-fold dilutions. NSC279836 exhibited a full inhibition of IN phosphorylation at the highest dose of 300 µM with an apparent IC_50_ in the low micromolar range (Figure 4c). Once more, a reproducible increase in GCN2 auto-phosphorylation was associated with this inhibition. Consistent with previous results, NSC758487 significantly affected IN phosphorylation without reaching full inhibition even at the highest dose of 300 µM (Figure 4d). Regarding NSC756644, a complete inhibition of both IN phosphorylation and GCN2 auto-phosphorylation could be observed only at a concentration of 300 µM (Figure 4e). On the other side, NSC180973 was a potent inhibitor of GCN2 with a full inhibition of both IN phosphorylation and GCN2 auto-phosphorylation at concentration above 33 µM and an apparent IC_50_ in the low micromolar range (Figure 4f).

**Figure 5:**
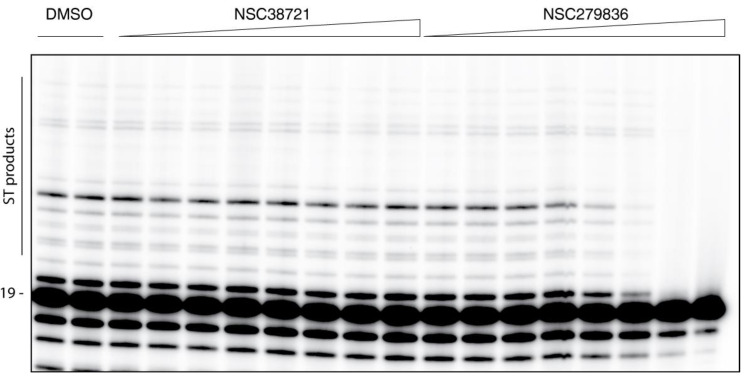
Effect of modulators on IN strand transfer activity. In this assay, only IN was present using serial dilutions of molecules from 100 µM to 45.7 nM following a 3-fold decrease. This representative image shows an inactive compound NSC38721 in parallel to an active compound, NSC279836. The first two lanes correspond to a DMSO control plus IN.

Finally, we tested all nine molecules for their ability to inhibit the IN ST activity in vitro. In this assay, only IN is present and any impact of the molecule would be associated to a direct effect of the molecule on IN. Except for NSC279836, none of the eight other molecules induced visible inhibition of the ST reaction (for example, see NSC38721, Figure 5). Serial dilutions of NSC279836 showed a typical dose-response inhibition of the ST activity (Figure 5). Full inhibition was observed at concentrations above 11 µM and the apparent IC_50_ was in the low-micromolar range.

## 3. Discussion

GCN2 is a target of growing interest in the context of cancer and neurodegenerative diseases. Playing a central role in the integrated stress response as a nutrient sensor, GCN2 is a multidomain protein with multiple interactions in the cell. The main path undertook to target GCN2 is via the development of active site inhibitors [18,21]. In previous studies, we identified GCN2 as a novel partner of the HIV-1 IN and showed that phosphorylation of IN was linked to a decrease in viral integration and replication [6,9,10].

In this work, we have developed an AlphaLISA assay to monitor the interaction between GCN2 and HIV-1 IN in vitro. Because the overall architecture of this complex is unknown, we were aiming at selecting small molecule ligands that might modulate this interaction to better study it. In a first attempt, we decided to perform a pilot screen using a limited number of molecules. The DTP oncology set is composed of 133 FDA-approved drugs with various targets (e.g., DNA repair enzymes, signal transduction) with partial chemical redundancy (for a complete list of tested molecules see Supplementary Materials). Altogether, we have selected nine compounds for further validation and preliminary characterization. Among them, five molecules inhibited the IN–GCN2 interaction while four were enhancing it.

Because GCN2 is a serine-threonine kinase, it is not surprising that the screen selected three kinase inhibitors. Interestingly, the approved oncology set contained 31 kinase inhibitors (mainly tyrosine kinase inhibitors, 24/31). NSC747599, also known as nilotinib, is a tyrosine kinase inhibitor targeting Bcr-Abl. It is used in cancer therapy to treat chronic myeloid leukemia. Interestingly, NSC758487 (ponatinib) was also selected. Ponatinib also targets Bcr-Abl but is less selective and inhibits other kinases, such as VEGFRs, FGFRs, TIE2 and Flt3. Finally, the third kinase inhibitor selected was NSC779217 (osimertinib). Osimertinib covalently binds mutant version of EGFR (another tyrosine kinase), close to the ATP binding pocket [22]. In the interaction assay, both ponatinib and nilotinib (the two Brc-Abl inhibitors) were stimulators while osimertinib (an EGFR inhibitor) was an inhibitor (Figure 3). Nonetheless, none of these three molecules actually inhibited GCN2 auto-phosphorylation in vitro (Figure 4); only ponatinib impacted IN phosphorylation. Altogether, these data would argue in favor for an activity of these three molecules directly on IN–GCN2 interaction without targeting the kinase activity of GCN2. A recent report showed that both nilotinib and ponatinib were able to inhibit SARS-CoV-2 replication in cell culture, probably through the binding of the Spike viral glycoprotein [23,24,25]. This would argue that these molecules have the potential to inhibit protein–protein interactions. Of note, there is already a precedent of tyrosine kinase being also a protein–protein inhibitor. Indeed, lavendustin B is a tyrosine kinase inhibitor that also inhibits the glucose transporter Glut1 and the IN-LEDGF interaction [26,27]. While other kinase inhibitors present in the library have not been tested in either the phosphorylation assay or the IN ST assay, more in depth characterization of the structure-activity relationship of this family of molecules could lead to a chemical lead for interfering with the IN–GCN2 interaction.

NSC766270, also known as Venetoclax (ABT-199) is an inhibitor of the anti-apoptotic Bcl-2 protein. ABT-199 competes with the binding of BH3-only proteins into a hydrophobic pocket. As Bcl-2 overexpression prevents reservoir cells from dying, ABT-199 has already been tested to reduce HIV reservoir [28]. Indeed, treatment in combination with Ixazomib was highly efficient at killing reservoir cells but also highly toxic in primary cells [29]. Here, we showed that ABT-199 is potent inhibitor of the IN–GCN2 interaction without affecting IN or GCN2 in vitro activities. A similar phenomenon was observed with NSC122758 (retinoic acid, also known as vitamin A). Unlike ABT-199, vitamin A induces cell differentiation and is known to inhibit telomerase in cells (telomeres shortening). Once again, this molecule was also reported to be antiviral against HIV [30] and a low-level of vitamin A in infected patients is associated with faster disease progression [31]. The antiviral effect has been associated with transcription, but also to the direct inhibition of the reverse transcriptase of HTLV-1 and HIV [32]. Still, it is unclear how ABT-199 and vitamin A can inhibit the IN–GCN2 interaction without affecting IN’s phosphorylation by GCN2. A probable explanation could be that the molecules are decreasing the overall stability of the complex without preventing its formation. Further characterization may be necessary to determine the mechanism of this inhibition. Nonetheless, given the relative harmlessness of vitamin A, a deeper analysis of the impact of the molecule on GCN2 and HIV would rationalize its use in parallel to targeted therapies, including cancer and viral infections.

NSC756644 (rucaparib), is a tricyclic indole inhibitor of poly(ADP-ribose) polymerase (PARP) proteins. In vitro, it promotes DNA-damaging agent’s activity as PARPs are involved in DNA damage-repair linked to single-strand breaks and protein adducts. In the approved oncology set, there were three PARP inhibitors, namely olaparib and niraparib (Supplementary Materials). Only rucaparib exhibited a stimulating effect on the IN–GCN2 interaction. Still, the effect of rucaparib on GCN2 phosphorylation was weak and only limited inhibition of IN and GCN2 phosphorylations were observed (Figure 4). While PARP proteins have already been described as important during HIV replication [33], PARP inhibitors may be modified to exacerbate their potency at stabilizing the IN-GCN2 complex.

NSC38721 (mitotane), is an anti-adrenocorticoid derived from dichlorodiphenyl trichloroethane. After metabolization, it covalently binds to adrenal proteins and strongly induces CYP3A4 [34]. Mitotane had no effect on either HIV-1 IN or GCN2 activities but strongly increased their interaction in the micromolar concentration range (Figure 4 and Figure 5). Judging by the simple chemical structure of mitotane with numerous halogen substitutions, it seems reasonable to assume that the molecule binds into a hydrophobic region of one or the other partner. Searching for derivatives in the DTP database resulted in the identification of 26 similar compounds, with 19 being readily available. Such further SAR study may help define the precise target (IN, GCN2 or both) as well as the estimation of the size of the targeted hydrophobic pocket.

NSC180973, tamoxifen citrate, is a nonsteroidal selective estrogen receptor modulator (SERM). Tamoxifen competes with the binding of estradiol leading to the inhibition of DNA synthesis and down regulation of PKC, among others. HIV replication was also found to be inhibited by tamoxifen through LTR transcription regulation [35]. Here, we found that tamoxifen was a potent inhibitor of the IN–GCN2 interaction without affecting IN activities. The molecule efficiently inhibited GCN2 activation, as well as IN’s phosphorylation. Thus, tamoxifen antiviral activity may be multimodal and warrants further cellular characterization.

NSC279836 (mitoxantrone) is an antibiotic, part of the anthracenedione family. Known to intercalate DNA and produce cross-link adducts, it inhibits DNA and RNA replication in cells. Mitoxantrone was shown to bind topoisomerase II and to induce DNA breaks [36,37]. In the oncology set, there were 10 topoisomerase inhibitors, 8 targeting Top2 and 2 targeting Top1. Except for etoposide and dexrazoxane, Top2 inhibitors decreased the IN–GCN2 interaction by 50 to 70% (data not shown). For Top1 inhibitors (topotecan and irinotecan) no effect was observed. Consistent with our results, mitoxantrone was found to inhibit HIV integrase in vitro [38,39], which might indicate a direct effect of the compound on IN. Nonetheless, one could argue that the intercalation property of the molecule could account for this inhibition rather than a direct interaction with IN. Still, it was also reported that the molecule bound hydrophobic structures, including tubulin, inhibiting microtubule polymerization [37]. Hence, mitoxantrone appears to be able to inhibit protein–protein interactions and, particularly, to bind hydrophobic regions that would favorable to binding to IN. In parallel, mitoxantrone was already known to activate eIF2α through PERK and GCN2 [40,41]. As we found a mitoxantrone inhibitor of the IN–GCN2 interaction, with a clear inhibition of IN phosphorylation and a reproducible increase in GCN2’s activation, it appeared that the molecule might actually bind directly GCN2 and possibly IN.

## 4. Materials and Methods

*Chemicals.* The approved oncology drugs set (AODVIII, 133 molecules) was obtained from the National Cancer Institute Developmental Therapeutics Program (DTP-NCI, NIH). The set is composed of two 96-well plates containing 20 µL at 10 mM in 100% DMSO. A-92 and MK-2048 were purchased from Axon MedChem and Selleckchem, respectively. All compounds were dissolved in 100% DMSO. Stock solutions (10 mM) were stored at −20 °C.

*Oligonucleotides.* Oligonucleotides were purchased from Integrated DNA Technologies, Inc. (Leuven, Belgium), purified on polyacrylamide gel through electro-elution and dissolve in water. 19T (GTGTGGAAAATCTCTAGCA) and 21B (ACTGCTAGAGATTTTCCACAC) correspond to the cleaved and non-cleaved strands specifically recognized by HIV-1 IN. Radiolabeling of 19T at the 5′-end was performed with [γ-^32^P] ATP (Perkin-Elmer, Villebon-sur-Yvette, France) using T4 polynucleotide kinase (Promega, Charbonnières-les-Bains, France), according to the manufacturers’ instructions. Unincorporated isotopes were removed using illustra microspin G-25 Columns (Cytiva life sciences, Amersham, Little Chalfont, United Kingdom). DNA duplexes were annealed using an equimolar ratio of the complementary strand 21B, heating to 95 °C, and slow cooling to room temperature.

*Enzymes.* GCN2 was purchased from SignalChem (EIF2AK4, cat. #E12-11G). This human recombinant protein was expressed as a truncated version (192-1024) in fusion after a glutathione S-transferase tag (approximate molecular weight of 132 kDa). Recombinant full-length IN was expressed in E. coli BL21 pLys (IPTG induction) and purified on a nickel chelating column, as described [42]. Integrity and purity were checked by direct Coomassie coloration of elution fractions on SDS-PAGE before dialysis to remove imidazole. Protein concentration was determined using a NanoDrop 2000 with the following parameters: molecular weight: 34 kDa, extinction coefficient ϵ = 50,460 mol^−1^cm^−1^L.

*IN–GCN2 interaction assay.* Protein–protein interactions were monitored using the AlphaLISA technology (Perkin Elmer) taking advantage of the GST and 6xHis tags of GCN2 and IN, respectively. Accordingly, AlphaScreen GSH Donor beads and AlphaLISA Nickel Acceptor beads were used (Perkin Elmer). Reactions were carried out at room temperature by incubating GCN2 with drugs or DMSO for 5 min before addition of AlphaLISA Nickel Acceptor beads. After 5 min, IN was added to the reaction mixture and incubated for 20 min before adding the AlphaScreen GSH Donor beads. This interaction assay was performed in conditions similar to the in vitro phosphorylation assay, already described [10], using 25 nM GCN2 and 700 nM (except indicated otherwise) in a reaction buffer containing 50 mM Tris-HCl pH 7.5, 40 mM NaCl, 1 mM dithiothreitol (DTT), 0.05% Tween 20, 0.1% bovine serum albumin (BSA), 10 µg/mL of each donor and acceptor beads. Alpha signal was measured using an EnSight plate reader (Perkin Elmer) 120 min after the addition of the latest component of the reaction (unless indicated otherwise).

*In vitro phosphorylation assay.* Phosphorylation of IN (700 nM) was carried out using 25 nM of GCN2 in a reaction buffer containing 10 mM Tris-HCl pH 7.5, 50 mM NaCl, 15 mM MgCl_2_, 7.5 mM MnCl_2_, 10 mM DTT, 0.005% Nonidet P-40, 100 µM ATP, 50 µCi/µL of [γ-^32^P]-ATP (3000 Ci/mmole, Perkin Elmer). Reactions (20 µL) were stopped after a 60 min incubation at 30 °C by adding 10 µL of loading buffer [80 mM Tris-HCl pH 6.8, 100 mM DTT, 10% glycerol, 1% sodium dodecyl sulfate (SDS) and 0.05% bromophenol blue]. Samples were then loaded onto a 10% denaturating SDS-PAGE. After protein separation (120 V for 120 min) the gel was stained using Coomassie Brilliant Blue R-250. Autoradiography was performed using an imaging plate (Fujifilm, Tokyo, Japan) and images were obtained with a FLA-5000 Imaging System (Fujifilm).

*Integrase reactions.* IN reactions were carried out by adding molecules or an equivalent volume of 100% DMSO (dimethyl sulfoxide, used as the drug solvent) to a mixture of 20 nM duplex DNA (19T/21B) and 400 nM IN in 50 mM MOPS pH 7.2, 7.5 mM MgCl_2_, and 14 mM 2-mercaptoethanol. Reactions were performed at 37 °C for 1 h and quenched by addition of an equal volume (10 µL) of loading buffer [formamide containing 1% SDS (sodium dodecyl sulfate), 0.25% bromophenol blue, and xylene cyanol]. Reaction products were separated in 16% polyacrylamide denaturing sequencing gels. Dried gels were visualized using a FLA-5000 imaging system (Fujifilm). Densitometric analyses were performed using the ImageQuant 5.1 software (GE Healthcare, Chicago, IL, USA) and data analyzed using Prism 6.05 software (GraphPad, San Diego, CA, USA).

## 5. Conclusions

In this study, we successfully developed an in vitro assay to monitor the IN–GCN2 interaction using AlphaLISA. Using the well-characterized FDA-approved oncology set from the DTP, we showed that the IN–GCN2 interaction can be modulated by small-molecule ligands. Among the 133 molecules, five were inhibitors and four were stimulators of the interaction at steady state. Interestingly, different kind of molecules were selected, from kinase active-site inhibitors to oestrogen-receptor modulators. Although deeper in vitro and cellular characterization is needed, these modulators may be useful tools for structural studies of IN and/or GCN2, as well as for determining the architecture of the complex. Ultimately, these molecules may be useful for deciphering the role of GCN2 during retroviral integration and more generally its implication in other diseases such as cancer and neuro-degenerative disorders. Altogether, this pilot screen demonstrates that it is possible to target the IN–GCN2 interaction in vitro and the screening of larger and more diverse chemical libraries is in progress to uncover original lead compounds.

## Supplementary Materials

The following are available online. A comprehensive table of the DTP oncology drugs set including NSC number, name and molecular weight.

## Sample Availability

Samples are not available due to being perishable goods.
